# Determination of *Baylisascaris schroederi* Infection in Wild Giant Pandas by an Accurate and Sensitive PCR/CE-SSCP Method

**DOI:** 10.1371/journal.pone.0041995

**Published:** 2012-07-27

**Authors:** Wenping Zhang, Shangmian Yie, Bisong Yue, Jielong Zhou, Renxiong An, Jiangdong Yang, Wangli Chen, Chengdong Wang, Liang Zhang, Fujun Shen, Guangyou Yang, Rong Hou, Zhihe Zhang

**Affiliations:** 1 The Sichuan Key Laboratory of Conservation Biology on Endangered Wildlife, Chengdu Research Base of Giant Panda Breeding, Chengdu, Sichuan, People’s Republic of China; 2 The Sichuan Key Laboratory of Conservation Biology on Endangered Wildlife, College of Life Science, Sichuan University, Chengdu, People’s Republic of China; 3 College of Life Sciences, Southwest Forestry University, Kunming, People’s Republic of China; 4 College of Animal Science, Sichuan Agricultural University, Ya’an, People’s Republic of China; 5 Tangjiahe National Nature Reserve, Guangyuan, People’s Republic of China; The George Washington University Medical Center, United States of America

## Abstract

It has been recognized that other than habitat loss, degradation and fragmentation, the infection of the roundworm *Baylisascaris schroederi* (*B. schroederi*) is one of the major causes of death in wild giant pandas. However, the prevalence and intensity of the parasite infection has been inconsistently reported through a method that uses sedimentation-floatation followed by a microscope examination. This method fails to accurately determine infection because there are many bamboo residues and/or few *B. schroederi* eggs in the examined fecal samples. In the present study, we adopted a method that uses PCR and capillary electrophoresis combined with a single-strand conformation polymorphism analysis (PCR/CE-SSCP) to detect *B. schroederi* infection in wild giant pandas at a nature reserve, and compared it to the traditional microscope approach. The PCR specifically amplified a single band of 279-bp from both fecal samples and positive controls, which was confirmed by sequence analysis to correspond to the mitochondrial COII gene of *B. schroederi.* Moreover, it was demonstrated that the amount of genomic DNA was linearly correlated with the peak area of the CE-SSCP analysis. Thus, our adopted method can reliably detect the infectious prevalence and intensity of *B. schroederi* in wild giant pandas. The prevalence of *B. schroederi* was found to be 54% in the 91 fecal samples examined, and 48% in the fecal samples of 31 identified individual giant pandas. Infectious intensities of the 91 fecal samples were detected to range from 2.8 to 959.2 units/gram, and from 4.8 to 959.2 units/gram in the fecal samples of the 31 identified giant pandas. For comparison, by using the traditional microscope method, the prevalence of *B. schroederi* was found to be only 33% in the 91 fecal samples, 32% in the fecal samples of the 31 identified giant pandas, and no reliable infectious intensity was observed.

## Introduction

The giant panda (*Ailuropoda melanoleuca*) is one of the world’s most widely recognized endangered species. It has been estimated that only 1600 individuals survive in the wild, living in six isolated mountain ranges within China [1]. Although habitat loss, degradation and fragmentation are the main threats to wild giant pandas [2], there is growing recognition that the infection of a parasitic roundworm called *Baylisascaris schroederi* is one of the major causes of death in the species [3]. This is confirmed by a report stating that during the period of 2001 to 2005; about 50% of deaths in wild giant pandas were caused by the parasite infection [3], [4].

The infection of *B. schroederi* in giant pandas was first reported by McIntosh [5]. *B. shroederi* usually infects the intestines of giant pandas, and can cause intestinal obstruction, inflammation, and even death [3]. According to a postmortem examination of 11 wild giant pandas, the intensity of adult roundworms was found to range from 1 to 619, where hundreds of adult roundworms can choke some of the pipelines linking the intestine and the stomach [6].

Overall prevalence of roundworm infection was reported to be 74.3%, and there were significant differences among habitats [7]. Wu et al. believed that wild giant pandas that lived in low elevation mountains should be more susceptive to the roundworms than those living in high elevation mountains because *B. schroederi* eggs can develop faster in high temperatures than in low temperatures [8]. However, Lai et al. reported that the overall prevalence of *B. schroederi* infection was 56%, and no differences were found among habitats [9]. In another report, the prevalence of *B. schroederi* infection was 54%, and no significant variation was found between any pair of mountains when authors only considered the infected giant panda individuals [4]. Nevertheless, differences in the prevalence were observed when all fecal samples were considered [4]. Although quantitative identification of the parasite infection is important for both management and application of appropriate therapy, only one study [4] has considered the intensity *B. schroederi* eggs in giant pandas.

The reasons for these inconsistent reports are not clear. However, in all these studies, the determination of parasitic infection was performed by using a technique of sedimentation-floatation followed by examination under a light microscope (sedimentation-floatation/microscope technique). In our own study, the data showed that this method is relatively poor in accurately determining parasite prevalence when there were many undigested bamboos or few *B. schroederi* eggs in fecal samples. This method is also inaccurate in determining the infectious intensity. Therefore, it is necessary to find another method that can overcome the shortcomings of the sedimentation-floatation/microscope technique in determining the prevalence and intensity of *B. schroederi* in giant pandas.

In this present report, we adopted a new method that used PCR and capillary electrophoresis combined with a single-strand conformation polymorphism analysis (PCR/CE-SSCP) to determine the infection of *B. schroederi* in wild giant pandas at the Tangjiahe National Nature Reserve in P.R. China. To compare the efficiency of our PCR/CE-SSCP method, analysis using the sedimentation-floatation/microscope technique was also performed in the study.

## Materials and Methods

### Specimen Collection

According to a survey [10], there are three subpopulations of wild giant pandas at the Tangjiahe National Nature Reserve living in the Hongshihe, Motianling, and Luoyigou mountain habitats, respectively. Collection of wild giant panda fecal samples was carried out during the period of 2009 to 2010 at the reserve.

Sample locations were recorded by GPS, and mapped with Arcview 3.2a when feces of the giant pandas were found. Up to 5 grams of fecal matter was extracted from the outer layer, and stored in 100% ethanol for individual identification of giant pandas. Then, in the same location, about 200 grams of the feces were crushed and stored in vials of 100% ethanol for examination of *B. schroederi* eggs. To avoid duplicate samples from one individual animal, additional feces within 500 m^2^ of a sample were not collected.

**Figure 1:**
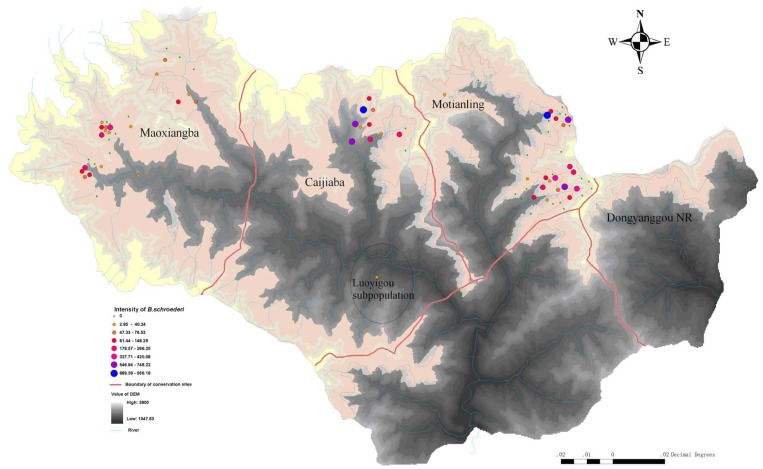
Geographical distribution of the collected samples at the Tangjiahe National Nature Reserve. The infectious intensity of *B. schroederi* in giant panda feces was determined with the PCR/CE-SSCP method.

In this way, a total of 91 fecal samples in which 32 from Hongshihe, 2 from Luoyigou, and 57 from Motianling, were collected respectively (Fig. 1 and Table 1). All activities followed the legal requirements and institutional guidelines set out by the government of P.R. China.

**Table 1:** Prevalence and intensity of *B. shroederi* infection determined by the PCR/CE-SSCP method and sedimentation-floatation/microscope technique.

Subpopulation			Hongshihe	Motianling	Luoyigou	Total
No. of faecal samples			32	57	2	91
No. of identified individuals			16	15	–	31
Microscopic method	Prevalence (%) of all samples		33(10/30)	35(20/57)	–	33(30/91)
	Prevalence (%) of identified individuals		25(4/16)	40(6/15)	–	32(10/31)
	Intensity		–	–	–	–
PCR/CE-SSCP method	Prevalence (%) of all samples§		59(19/32)	51(29/57)	50(1/2)	54(49/91)
	Prevalence (%) of identified individuals§		44(7/16)	53(8/15)	–	48(15/31)
	Intensity of all samples	Mean	88.3**	284.2**	3	181.2
		VMR*	158.8	428.5	–	389.4
	Intensity of identified individuals	Mean	150.6	355.1	–	259.7
		VMR*	234.3	500.4	–	444.7

§ The positive samples checked by PCR were amplified successfully at least twice in one of five template concentration.

* VMR: the variance-to-mean ratio calculated by using Quantitative Parasitology 3.0 programe.

** Bootstrap test, *P*-value  = 0.0145.

### Individual Giant Panda Identification

Ten giant panda special microsatellite loci described by Yang et al. [11] were used in our study. PCR products were resolved by using an ABI 3100 automated sequencer (Applied Biosystems Inc.), and analyzed by using the GenoTyper Analysis Software (ver. 3.7, Applied Biosystems Inc.). Reliable genotypes of each fecal sample were obtained according to the methods described by Yang et al. [11] and Solberg et al. [12]. GIMLET [13] was used to estimate the probability of full-sib or unrelated pairs of giant pandas with an identical multi-locus genotype.

### Identification of *B. schroederi* with Sedimentation-floatation/microscope Method

About 100 grams of each sample were examined for *B. schroederi* eggs by using the sedimentation-floatation/microscope technique as described by Lai et al. [8]. Each sample was first mixed with 250 ml of distilled water, and bamboo residues that were more than 1 cm in length were discarded from the mixture with forceps. The remaining mixture was then filtered through a wire mesh of 370-µm aperture. After 30 min of sedimentation, supernatant was siphoned off, and the sediment was checked for *B. schroederi* eggs under a light microscope. If no eggs were detected in the sediment, the remainder of the sediment was mixed with saturated NaCl solution to further examine following the method of Lai et al. [8].

### DNA Extraction

The remaining 100 grams of each sample was treated by mixing with distilled water, cleaning the bamboo residues and filtering through wire meshes under sterile conditions. Afterwards, the filtrates were centrifuged at 5000 rpm for 10 min. DNA in the sediment was extracted by using a QIAamp Stool Mini Kit (Qiagen Inc.) according to the manufacturer’s instructions with a slight modification. Briefly, 200 mg of sedimentation were transferred to a tube containing 1.6 ml of ASL buffer with 7.5 µM of CaCl_2_ and 40 mM of DTT, and boiled for 30 min. Then, another 1.6 ml of ASL buffer with 1 mg/ml of proteinase K was added, heated to 70°C for 30 min, and incubated at 55°C overnight. On the following day, the mixture was centrifuged at 12000 rpm for 2 min and the supernatant was transferred to a new sterile tube for subsequent steps in the kit process. Finally, DNA was eluted by 200 µL of AE buffer. In addition, two negative controls were used: one was water and another was a stool sample of a healthy captive giant panda during this process of DNA extraction.

At the same time, two positive controls of DNA were obtained. One control was extracted by using the above protocol from a fecal sample of a captive giant panda containing *B. schroederi* eggs that was confirmed by the sedimentation-floatation/microscope technique, and another control was extracted by using standard protocols [14] from an adult *B. schroederi*.

### Primers and Amplification

It was found that there are four sequences of mitochondrial *cytochrome coxidase subunit* II (COII) gene of *B. schroederi* in GenBank (FJ890506, FJ890511, FJ890512, and FJ890513, Genbank no) with three haplotypes. This suggested that COII could be used to perform the genetic assessment of *B. schroederi*. Base upon alignment of the complete mitochondrial genomes of *B. schroederi*, *B. ailuri* and *B. transfuga* isolated from giant pandas, red pandas and polar bears, respectively [15] and the four sequences of the mitochondrial *cytochrome coxidase subunit* II (COII) gene, primers PASCARIS-COII F 5′-HEX-TGTATTATTATGGTTTGATGAA-3′ and PASCARIS-COII R 5′-TCATAGCATCCAACTTAATAGAC-3′ were designed to amplify a 279-bp fragment of the gene. Unlabeled and 5′-labeled primers with a hexachloro derivative of fluorescein (HEX primers) were synthesized by Invitrogen Inc. (Shanghai, China).

The PCR consisted of 15 pmol primers, 200 µM each dNTP, 3.5 µl 1 µg/µl BSA, 0.5 U of AmpliTaq Glod™ DNA polymerase (Applied Biosystems Inc.), and 2.1 µl of 25 mM MgCl_2_ in a 15 µl total volume. In order to determine the correlation between the peak area of CE-SSCP and the amount of genomic DNA, 5 different DNA template volumes (0.5 µl, 1.0 µl, 1.5 µl, 2.0 µl, and 2.5 µl) for each fecal sample were applied. For the control sample of adult *B. schroederi*, 2 ng, 4 ng, 6 ng, 8 ng, and 10 ng of DNA were added. For all PCR runs, both positive and negative controls were included, and all samples were run in triplicates for each template concentration. PCR were performed on a GeneAmp® 9700 thermal cycler (Applied Biosystems Inc.) with the following conditions: 10 min at 95°C followed by 20 cycles of 30 s at 94°C; 30 s at stepwise lowering of the annealing temperature from 55°C to 46°C and 30 s at 72°C; 10 cycles of 30 s at 90°C, 30 s at 49°C, and 30 s at 72°C. The final extension time was 30 min at 72°C. Following PCR, 6 µl of the product was electrophoresed on 1.5% agarose gels and DNA was visualized in the gel by the addition of ethidium bromide as described previously [13].

### CE-SSCP Analysis and Sequencing

PCR products from the adult *B. schroederi* and one or two positive PCRs per fecal sample were analyzed with the CE-SSCP as described by Park et al. [16] and Shin et al. [17]. Park et al. [16] found that high concentration DNA templates might result in nonlinear correlation between the peak areas of CE-SSCP and DNA amounts. Thus, the selected positive PCRs for the CE-SSCP were low volume DNA templates.

In addition, PCR products from the adult *B. schroederi* and 6 stochastic positive samples were obtained by PASCARIS-COII F/R. After these products were electrophoresed on 1.5% agarose gels, the bands whose sizes located at 200–300 were cut and reclaimed. Then, the targets were purified using a gel extraction mini kit (Watson Biotechnologies) and bidirectional sequenced with an ABI 310 DNA sequencer.

### Statistical Analysis

The prevalence and intensity of *B. schroederi* infection in wild giant pandas were analyzed by using the Quantitative Parasitology 3.0 software program [18]. The comparison of prevalence and intensity between wild giant panda subpopulations was performed with a Chi-square test and Bootstrap test, respectively, as recommended by the software [18]. Correlation between the amount of DNA template and the peak area of CE-SSCP was analyzed using a linear regression analysis. Statistical analysis was performed using the SPSS software package (ver. 14, Abacus Concepts).

## Results

### Individual Number

Due to the poor quality of fecal DNA, only 34 out of the 91 fecal samples were successfully performed for individual identification with the 10 microsatellite loci under the methods of Yang et al. [11] and Solberg et al. [12]. Only 31 individual giant pandas were identified from the 34 fecal samples: 16 from the Hongshihe subpopulation and 15 from the Motianling subpopulation (Table 1). GIMLET analysis demonstrated that the combination of the 10 chosen loci produced identical genotypes of full siblings by chance with a probability of 2.4×10^−5^, which is consistent with the report by Yang et al. [11].

### Validation of the PCR/CE-SSCP Method

Specificity of the PCR product in the adult *B. schroederi* completely matching the corresponding portion of FJ890506 (Genbank no.) was confirmed by using a sequence analysis. The sequence of 279-bp fragment without terminator was translated into 93 amino acids by DNAMAN (version 4.0, 1998). The results of Blast in GenBank show the sequence is from the mitochondrial genome of *B. schroederi* and only one variable site exists between our sequence and the matching corresponding portion of the complete mitochondrial genomes of *B. schroederi* reported by Xie et al [15].

**Figure 2:**
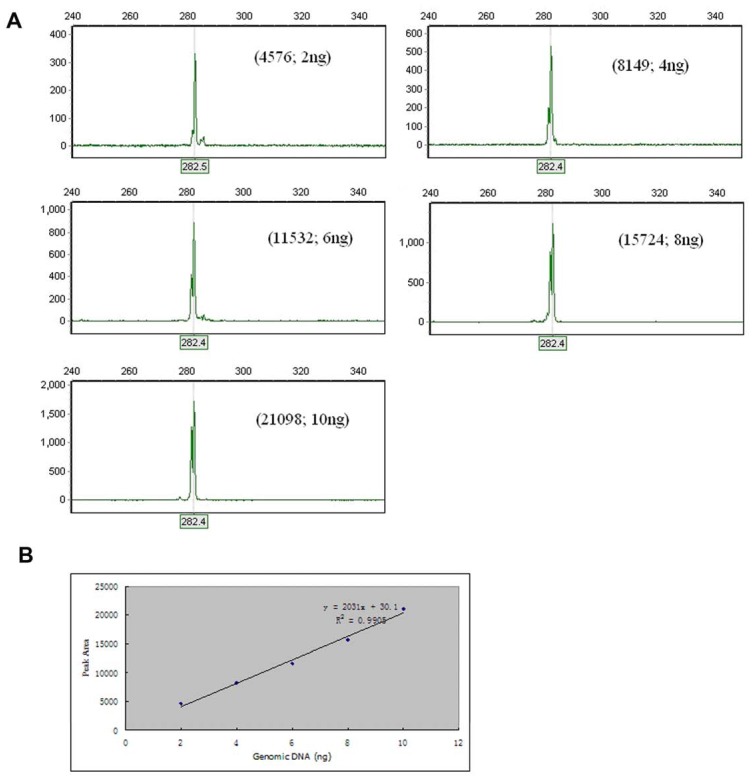
The relationship between template DNA amounts and the peak area of CE-SSCP. (A) Electropherogram of PCR products generated from different amounts of genomic DNA (2 ng, 4 ng, 6 ng, 8 ng, and 10 ng). The size is indicated on the x-axis, and fluorescence intensities are indicated on the y-axis. The numbers in parentheses indicates the peak area and DNA amount, respectively. (B) Correlation between the peak area and the amount of genomic DNA. Only one peak area in the triplicate of each DNA template amount was showed here. X- and Y-axes represent the amount of genomic DNA and peak area, respectively. R is the correlation coefficient.

The electropherogram of CE-SSCP showed a single major peak at size 282 (Fig. 2A), and there was no significant variation in peak area in the triplicate of each DNA template amount, and template DNA amounts were significantly correlated with the peak area of CE-SSCP (*Y*  = 30.1+2031*X*, *R^2^* = 0.9905, *P*<0.0001, Fig. 2B).

**Figure 3:**
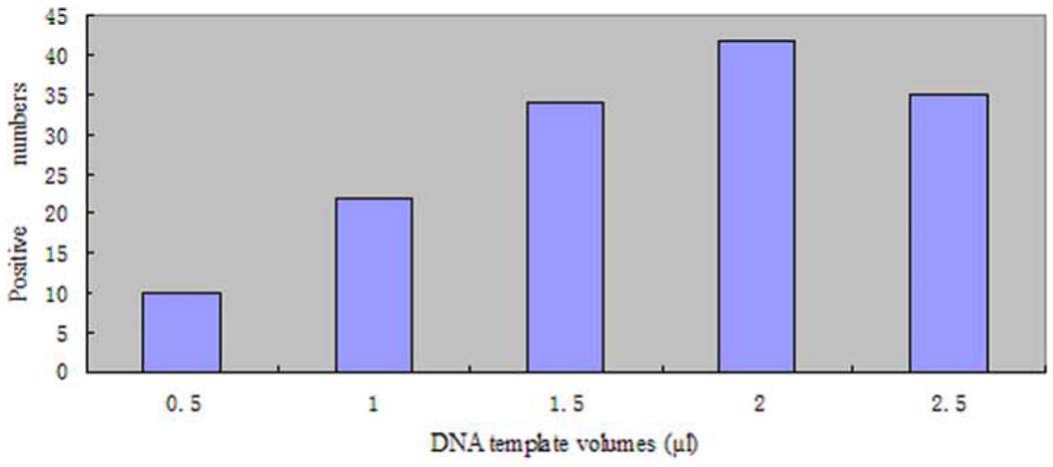
Correlation between DNA template volumes and number of positive samples. Here, the positive samples of one volume were amplified successfully at least twice in the corresponding template volume.

In the analysis of fecal samples, the PCR also produced a single band of 279-bp, and no variation in size existed in the PCR products on agarose gels, which was also observed by CE-SSCP analysis. Sequence analysis of the 6 stochastic positive samples further confirmed that they completely matched the corresponding portion of FJ890506. Moreover, increasing template DNA volumes from 0.5 to 2.0 µl increased the number of positive samples, while 2.5 µl of template DNA did not amplify more positive numbers than 2.0 µl (Fig. 3).

These results validated the specificity of the PCR method in detection of *B. schroederi* infection in giant pandas, and revealed that there is only one mitochondrial haplotype of *B. schroederi* in the infected wild giant pandas at the reserve.

### Prevalence and Intensity of *B. schroederi* Infection Determined by the PCR/CE-SSCP Method and Sedimentation-floatation/microscope Method

Table 1 shows that *B. schroederi* eggs were found in 30 out of the 91 samples (33%) by using the sedimentation-floatation/microscope technique, in which 10 were from the Hongshihe subpopulation and 20 were from the Motianling subpopulation. For the 31 identified giant pandas, 10 (32%) were found to be infected by the parasite in which 4 were from the Hongshihe and 6 were from the Motianling subpopulations. No reliable infectious intensity could be determined by using the microscope technique.

For comparison, by using the PCR method, *B. schroederi* infection was found in 49 out of 91 fecal samples (54%), in which 1 was from the Luoyigou, 19 were from the Hongshihe and 29 were from the Motianling subpopulations, respectively (Table 1). The 49 samples included 30 positive samples determined by the sedimentation-floatation/microscope technique.

The peak area of CE-SSCP in the 49 samples ranged from 1,017 to 128,013 fluorescent intensity units. Overall infectious intensity, calculated as units per gram feces, ranged from 2.8 to 959.2 (Fig. 1) with a mean of 181.2 and a variance-to-mean ratio of 389.4 (Table 1). The infectious intensity of the Hongshihe subpopulation ranged from 2.8 to 420.1 with a mean of 88.3 and a variance-to-mean ratio of 158.8. The infectious intensity of the Motianling subpopulation ranged from 5.5 to 959.2 with a mean of 248.2 and a variance-to-mean ratio of 428.5. The one positive sample from the Luoyigou subpopulation had a value of only 3.3 units per gram feces (Fig. 1 and Table 1). No significant difference in the prevalence was found between the Hongshihe and the Motianling subpopulations (Chi-square, *P*-value  = 0.440), but there was a significant difference in the mean intensity between the two areas (Bootstrap test, *P*-value  = 0.0145) when all fecal samples were considered.

There was a large difference in the intensity detected by different methods (Table 2). The mean intensity of samples that were positive for both the conventional microscopy technique and the PCR/CE-SSCP was 249.7; however, the mean intensity of samples that were positive only for the PCR/CE-SSCP method was 73.1. The difference was highly significant (*P*  = 0.005). The results suggested that PCR/CE-SSCP method may be more sensitive than the conventional method.

**Table 2:** The difference of intensity of *B. shroederi* in different samples determined by different methods.

	No. of faecal samples	Mean intensity	*Standard deviation*
Determined by both microscopic examination and PCR/CE-SSCP	30	249.7*	*272.6*
Determined by only PCR/CE-SSCP method	19	73.1*	*94.1*

* Bootstrap test, *P*-value  = 0.005.

For the 31 individually identified giant pandas, 15 were infected with *B. schroederi* (48%), in which 7 were from the Hongshihe and 8 were from the Motianling subpopulations (Table 1). Overall mean infectious intensity was 259.7 units per gram faces with a variance-to-mean ratio of 444.7. The infectious intensities of the infected animals from Hongshihe ranged from 4.8 to 420.1 with a mean of 150.6 and a variance-to-mean ratio of 234.3. For the Motianling subpopulation, the infectious intensities ranged from 47.4 to 959.2 with a mean of 355.1 and a variance-to-mean ratio of 500.4 (Table 1). No significant differences in prevalence (Chi-square, *P*-value  = 0.594) and mean intensity (Bootstrap test, *P*-value  = 0.1840) were found between the two subpopulations.

## Discussion

In this study, we adopted a method that combined PCR and CE-SSCP analysis to detect the infectious prevalence and intensity of *B. schroederi* in wild giant pandas at the Tangjiahe National Nature Reserve. Although only one mitochondrial haplotype was found in *B. schroederi* of giant pandas in this reserve based on CE-SSCP and sequences analysis, it was necessary to use CE-SSCP to detect PCR products amplified with fecal DNA and PASCARIS-COII F/R. This was because that the pool of *B. schroederi* eggs in feces of one host animal might contain different mtDNA haplotypes and PASCARIS-COII F/R could amplify simultaneously these haplotypes. The alignment of products of PASCARIS-COII F/R with HQ671081, FJ890511, FJ890512, and FJ890513 (Genbank no) contained some variable sites.

By using our method, the sensitivity of detecting the prevalence of *B. schroederi* infection increased dramatically when compared to the traditional sedimentation-floatation/microscope examination technique. Moreover, our method provides a quantitative measurement of the infectious intensity, which is difficult to determine by using the microscope approach.

Giant pandas consume about ∼12.5 kg of bamboo each day [19], and only ∼17% of the dry matter with low digestion coefficients for bamboo hemicelluloses and celluloses [20]. These undigested bamboo residues may disturb the visual field of the microscope, and hamper the separation of the *B. schroederi* eggs from the feces in the sedimentation-floatation equipment. Our PCR-method does not include sedimentation-floatation steps. Thus, it can overcome the shortcomings associated with the sedimentation-floatation/microscope examination technique. Moreover, PCR is an extremely sensitive method because it allows marker genes of specific pathogens present at extremely low levels to be detected by exponential amplification of the target [21], [22]. Our data showed that *B. schroederi* eggs are very difficult to find when using the traditional microscope examination if there are few eggs in the fecal samples (Table 2).

It is well-known that the use of fecal material as noninvasive sources of genetic material has a number of problems, such as co-purifying contaminants and low amounts of DNA [23]. In order to overcome these problems, we ran the samples at different input DNA volumes. The results showed that increasing the template DNA volumes could increase the number of positive samples when the template DNA volume ranged from 0.5 to 2.0 µl. This suggests that the different input DNA volumes for one sample should be necessary to determine reliable prevalence of parasite infection.

Quantitative evaluation of *B. schroederi* infectious intensity is important in understanding the ecological characteristics of endangered species, and improving their management [24]. We found that the intensity of *B. schroederi* was irreproducible among multiple repeated examinations of a single sample under microscope examination. This is consistent with observations made by Yang et al. (unpublished data, 2012). Moreover, many co-purifying contaminants in DNA templates make it impossible to obtain precise DNA concentration of *B. schroederi* in noninvasive samples. The CE-SSCP analysis is a highly sensitive and reproducible separation method for nucleic acids with widespread applications [25]. Park et al. [15] and Shin et al. [16] demonstrated that CE-SSCP analysis could be used for precise quantification of mRNA and DNA. Our study showed that the amounts of genomic DNA in *B. schroederi* were linearly correlated with the peak areas of CE-SSCP. Therefore, the use of the peak area of CE-SSCP per gram of infectious feces to evaluate the intensity of parasites was a reliable methodology. However, we did not observe a linear correlation between the peak area of CE-SSCP and the number of *B. Schroederi* eggs in this study. In order for our method to become a standard, further studies must be performed.

The prevalence of *B. schroederi* at the Tangjiahe National Nature Reserve as determined by our method was similar to that reported by Zhang et al. [4] across all mountain habitats. However, we found a significant difference in the mean intensity between the Hongshihe and the Motianling subpopulations when all fecal samples were considered (Table 1 and Fig. 1). Nielsen et al. [26] found significant linear relationships between *Strongylus vulgaris* egg numbers and cycle of threshold (*C*_t_) values based on a fluorescence-based quantitative PCR assay. Thus the number of eggs is directly proportional to the amount of extracted DNA. Our data showed also significant linear relationships between the amounts of genomic DNA in *B. schroederi* and the peak areas of CE-SSCP. Thus, the mean *B. schroederi* egg counts in the Motianling population were more than that in the Hongshihe subpopulation when all fecal samples were considered. In another report, Nielsen et al. [27] also found the fecal egg counts correlates to a higher burden of adult *Parascaris equorum* infection in horses. These suggested that the mean burden of adult *B. schroederi* infection in Motianling subpopulation might be higher than that in Hongshihe subpopulation.

The eggs of *B. schroederi* have been reported to be highly resistant to environmental degradation, and *B. shroederi* infection can persist in giant pandas for 1 to 2 years [3]. It has also been observed that the fertilized eggs of *B. schroederi* can develop into free-living infective stages rapidly, and then specifically infect other giant pandas [28]. Therefore, there is great need to survey the prevalence and intensity of *B. schroederi* infection for giant pandas at regular intervals. The PCR/CE-SSCP method developed by us should be a useful tool to monitor and compare the prevalence and intensity of *B. schroederi* among different habitats and the development of *B. schroederi* in one habitat, which will enable us to find severe infectious giant pandas and their habitats, thereby pay more attention to these individuals and habitats to perform routine surveillance of infectious intensity and use targeted strategic treatments.
